# Effect of *Bactrocera minax* (Diptera: Tephritidae) adult population density on its marginal distribution

**DOI:** 10.1093/jisesa/ieaf028

**Published:** 2025-05-16

**Authors:** Yibing Guo, Bo Xu, Cong Huang, Huimin Yang, Fulian Wang, Lianyou Gui, Guifen Zhang

**Affiliations:** Forewarning and Management of Agricultural and Forestry Pests, Hubei Engineering Technology Center, Institute of Entomological Science, College of Agriculture, Yangtze University, Jingzhou, Hubei, China; Forewarning and Management of Agricultural and Forestry Pests, Hubei Engineering Technology Center, Institute of Entomological Science, College of Agriculture, Yangtze University, Jingzhou, Hubei, China; State Key Laboratory for Biology of Plant Diseases and Insect Pests& Institute of Plant Protection, Chinese Academy of Agricultural Sciences, Beijing, China; Forewarning and Management of Agricultural and Forestry Pests, Hubei Engineering Technology Center, Institute of Entomological Science, College of Agriculture, Yangtze University, Jingzhou, Hubei, China; Forewarning and Management of Agricultural and Forestry Pests, Hubei Engineering Technology Center, Institute of Entomological Science, College of Agriculture, Yangtze University, Jingzhou, Hubei, China; Forewarning and Management of Agricultural and Forestry Pests, Hubei Engineering Technology Center, Institute of Entomological Science, College of Agriculture, Yangtze University, Jingzhou, Hubei, China; Forewarning and Management of Agricultural and Forestry Pests, Hubei Engineering Technology Center, Institute of Entomological Science, College of Agriculture, Yangtze University, Jingzhou, Hubei, China

**Keywords:** citrus fruit fly, distribution status, prediction and forecast

## Abstract

In order to explore the impact of insect density on the edge effect of *Bactrocera minax* (Diptera: Tephritidae) adult distribution in orange orchards, traps were set up in orchards with maggot infestation rates of 2%, 4%, and 20% to attract adults. The study compared differences in distribution between the side with noncitrus trees and the side farther away from them. The results showed that at lower insect densities (2% and 4% maggot infestation rates), the proportion of insect trap sites and the number of insects per trap on the side of the orchard adjacent to the trees were significantly higher than that on the side away from the noncitrus trees, additionally, the proportion of adults captured 10 to 20 m away from the side of noncitrus trees was also significantly higher than at other distances. However, at higher insect density (20% maggot infestation rates), there were no significant differences in the proportion of insect traps or the number of insects per trap between the adjacent and distant sides of the trees. Similarly, there were no significant differences in the proportion of adults captured at distances of 10, 20, 30, and 40 m away from the side of noncitrus trees in the orchard. In summary, at low insect density, *B. minax* adults exhibit a strong edge effect, concentrating on the side of the orchard with noncitrus trees, whereas at high insect density, they are evenly distributed throughout the orchard.

## Introduction

*Bactrocera minax* (Enderlein) (Diptera: Tephritidae), commonly known as the Chinese citrus fly, is identified as one of the most significant pests impacting citrus fruit (Chen and Xie 1955, Gong et al. 2019). The *B. minax* larvae consume the interior of the fruit, causing premature yellowing and early fruit drop leading to fruit loss and decay, this damage diminishes both food supply and economic loss, and threatens agricultural production (Wang and Lou 1995, Dorji et al. 2006, Wang 2019). *B. minax* is a univoltine insect with a prolonged adult stage (Wu 1958, Wang and Luo 1995). After emerging from the soil in the orange orchard, newly hatched male and female *B. minax* adults temporarily leave the orchard to feed on nectar, bird droppings, sooty mold fungi, and other resources found on nonhost plants. Once they reach sexual maturity, they return to the orchard to mate and lay eggs (Wang and Luo 1995, Luo et al. 2016).

Currently, eliminating *B. minax* adults through the use of food attractants is recognized as one of the most effective control methods (Zhang et al. 2007, Lan et al. 2009, Gong 2012, Yang et al. 2014). When food is abundant and unaffected by external factors, insects tend to remain close to food sources, leading to short-distance dispersal. However, when food becomes scarce or insect adaptability declines, they are more likely to disperse over greater distances (Shi et al. 2017). In addition to nutritional needs, the search for suitable reproductive environments, especially for egg-laying and mating sites, also plays a crucial role in insect dispersal (Dingle 1972, Holland et al. 2006). He et al. (2019) found that radar tracking studies show that newly emerged adults often leave citrus orchards to feed on chestnut male flowers outside the orchards, indicating that *B. minax* adults obtain nutrition from forest habitats outside the orchards. Similarly, Dong et al. (2014) reported that *B. minax* adults feed on nectar from nonhost plants, sooty mold mycelium, and bird droppings. This illustrates that the formation of within- and between-orchard movement patterns in *B. minax* populations is closely linked to the individual’s nutritional needs.

Additionally, density influences the distribution of insects in the field (Klomp 1964, Taylor 1984, Gray and Steffey 1998, Wang et al. 2002), and the distribution of different insects varies under different densities (Li et al. 2017, Yu et al. 2021). For example, species such as *Aphis spiraecola*, *Bemisia tabaci*, and *Spathius agrili* exhibit clustering across all densities (Wang and Yang 2005, Li et al. 2010, Wang et al. 2024). The aggregation intensity of *Bactrocera dorsalis* increases with higher densities (Lin et al. 2005, Liu et al. 2012). Therefore, understanding the effect of *B. minax* density on its marginal distribution in orchards under varying densities is crucial for effectively deploying traps for control purposes.

Studying and understanding the aggregation and distribution patterns of pests is crucial for assessing the extent of pest damage. This knowledge is vital for conducting field surveys, dynamically monitoring pest occurrences, and implementing effective prevention and control measures. However, there is limited research on whether insect density affects the distribution of *B. minax* outside orange orchards. The impact of varying insect densities on the marginal distribution of *B. minax* in field conditions remains unclear. Therefore, this study deployed traps in orange orchards under varying insect densities as measured by fruit infestation by larvae to investigate the aggregation of *B. minax* adults at the orchard edges. The research further clarified the occurrence, habitat, and distribution patterns of *B. minax* adults in orange orchards, and analyzed their dynamics under different insect densities. The goal is to provide a scientific basis for field prediction and effective prevention and control of *B. minax*.

## Materials and Methods

### Citrus Orchard Location

**Fig. 1.**
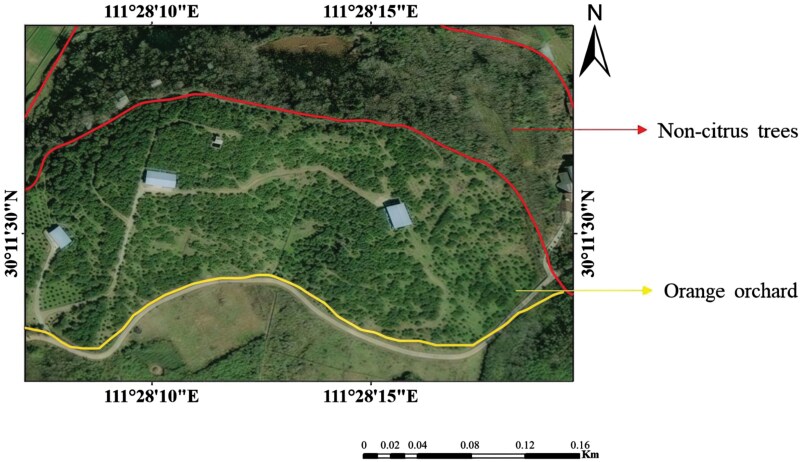
Satellite image of an orange orchard, with the red-bordered area representing non-citrus trees and the yellow-bordered area representing orange trees.

The citrus orchard investigated is located in Zhicheng town, Yidu city, Hubei province (30°15′N, 112°22′E), covering an area of 40,000 m^2^. It has a subtropical monsoon climate, with an average annual temperature of 16.7 °C, an average precipitation of about 1,212 mm, and a frost-free period of about 275 d. The orchard is situated on a hillside, with a noncitrus tree area to the north (Fig. 1).

### Trap Point Setup

Field surveys to assess *B. minax* maggot infestation rates were conducted in mid-August over 3 consecutive years. Using the parallel jump method, 30 points were selected, and 2 orange trees were examined at each point. From each tree, 30 citrus fruits were randomly collected from 4 directions (east, south, west, and north) to determine the number of fruits damaged by *B. minax*. Damaged fruits were those that turned yellow before ripening, exhibited yellow and red discoloration, or showed signs of being hardened or rotten. Over the 3 yr, the observed maggot infestation rates were 2%, 4%, and 20%, representing the annual insect densities.

**Fig. 2.**
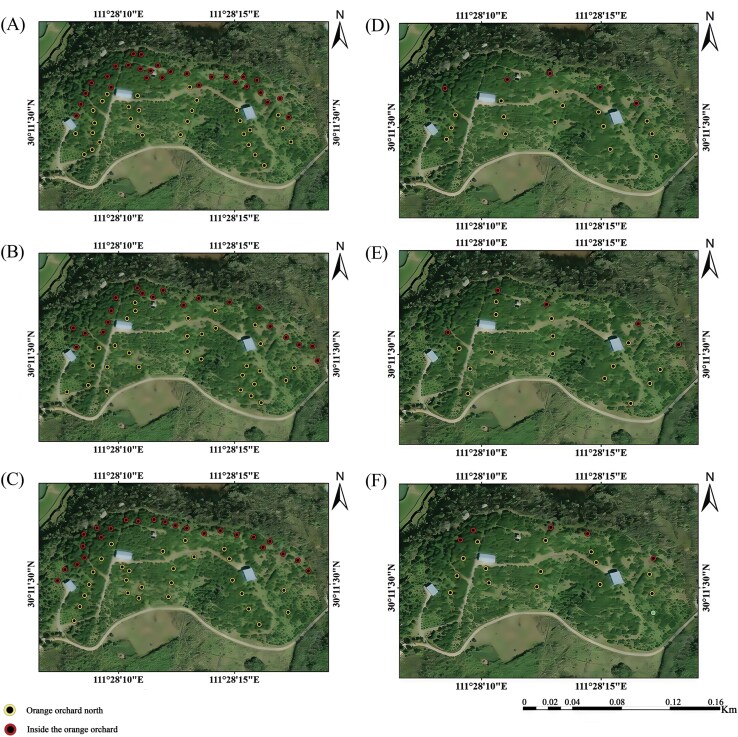
The distribution of survey points in citrus orchards at various infestation rates: A) 2%, B) 4%, and C) 20%. The proportion of insects at varying distances from noncitrus trees across different infestation rates: D) 2%, E) 4%, and F) 20%.

In the years with varying insect densities (2%, 4%, and 20%), a total of 58, 50, and 51 traps were deployed, respectively. Each trap contained a food attractant. Some traps were positioned in the northern part of the citrus orchard, near the noncitrus trees (28 traps for the 2% infestation rate, 20 traps for the 4%, and 24 traps for the 20%). The remaining traps were placed in the inner part of the orchard, away from the noncitrus trees (30 traps for the 2% infestation rate, 30 for the 4%, and 27 for the 20%) (Fig. 2A to C). For 3 consecutive years, from 17 May to 18 July, adult flies were collected at each trap approximately every 8 d, recording the number of male and female flies, and replacing the bait as needed. The longitude and latitude of each trap were recorded using a GPS device (Zhuolin Technology A8).

### Food Attractants

The experiment utilized edible citrus fruit fly bait produced by Jufeng Company, located in Yichang City, Hubei Province. For every 500 ml of poison bait, 3 to 4 liters of water were added, and the insecticide used was 24% Wanling water-soluble liquid (produced by Jiangmen pesticide factory, Guangdong province).

### Percentages of Traps with Adults

The trapping points in the northern and inner areas of the citrus orchard were randomly divided into 5 groups. The percentage of traps with adults in the northern area, relative to the total number of traps in the orchard, was recorded as the adult trapping percentage in the north. Similarly, the percentage of traps with adults in the inner area, relative to the total number of traps, was recorded as the adult trapping percentage inside the orchard.

### Investigation of Insect Density

The trapping points in the northern and inner parts of the orange orchard were randomly divided into 5 groups to calculate the insect density of *B. minax* adults. The insect density in the northern part of the orchard was determined by the ratio of the total number of insects trapped to the total number of trapping points in the north. Likewise, the insect density inside the orchard was calculated by the ratio of the total number of insects trapped to the total number of trapping points within the orchard.

### Investigation of the Proportion of Insects at Various Distances

In the northern part of the orange orchard, a trapping point adjacent to noncitrus trees (10 m from the trees) was selected as the reference point. Additional trapping points were selected within the orchard at distances of 20, 30, and 40 m from the noncitrus trees (Fig. 2D to F). The proportion of insect densities at different distances from the noncitrus trees to the total insect densities in the orange orchard was then calculated.

### Statistical Analyses

Data were analyzed using SPSS 23.0 (SPSS Inc., Chicago, IL, USA). The percentage of adult traps in the northern and inner areas of the orange orchard, as well as the insect density in these areas, were tested for normality at different insect densities (2%, 4%, and 20%). The Mann–Whitney *U* test was applied for comparisons between the 2 areas when the data did not follow a normal distribution, while the independent sample *t*-test was used when the data conformed to a normal distribution. The levene method was used to test the homogeneity of variance in the percentage of *B. minax* adults at varying distances from the noncitrus tree side under different insect densities (2%, 4%, 20%). Since the percentage of insect populations at different distances showed homogeneity of variance, one-way analysis of variance and the least significant difference test were used to assess the differences in *B. minax* adults’ percentage at varying distances from the noncitrus tree side under different densities.

## Results

### Percentages of Traps with Adults

**Fig. 3.**
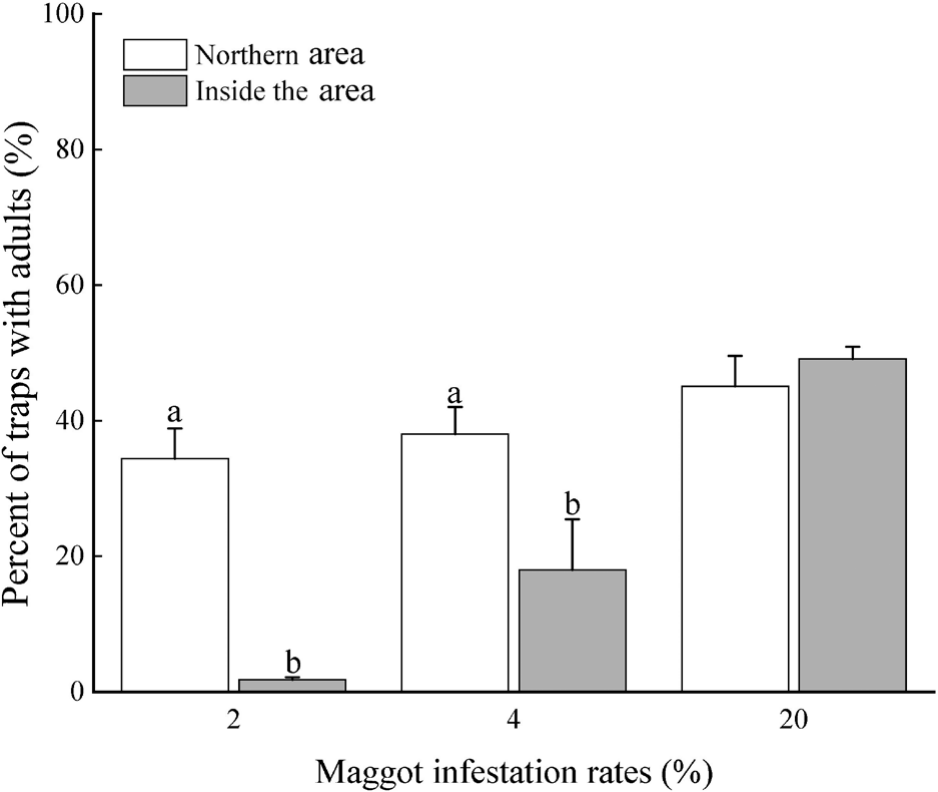
The percentage of traps with adults of *B. minax* in the northern and inner parts of the orchard under different infestation rates. Different lowercase letters indicate significant differences in proportion of traps with adults between the northern and inner parts of the orchard. The error bars in the figure represents standard errors (*P*<0.05).

When the maggot infestation rates were 2% and 4%, the percentages of traps with adults in the northern part of the orange orchard were 34.4% and 38.0%, respectively, which were significantly higher than those in the inner orchard (2% infestation rate: 1.8%, *U* = 0.000; *W* = 15.000; *Z* = −2.703; *P* = 0.008; 4% infestation rate: 7.5%, *U* = 0.500; *W* = 15.500; *Z* = −2.612; *P* = 0.008). However, at a maggot infestation rate of 20%, there was no significant difference in percentages of traps with adults between the northern and inner parts of the orchard (*U* = 6.500; *W* = 21.500; *Z* = −1.423; *P *= 0.222), with rates of 45.1% and 49.1%, respectively (Fig. 3).

### Insect Density

**Fig. 4.**
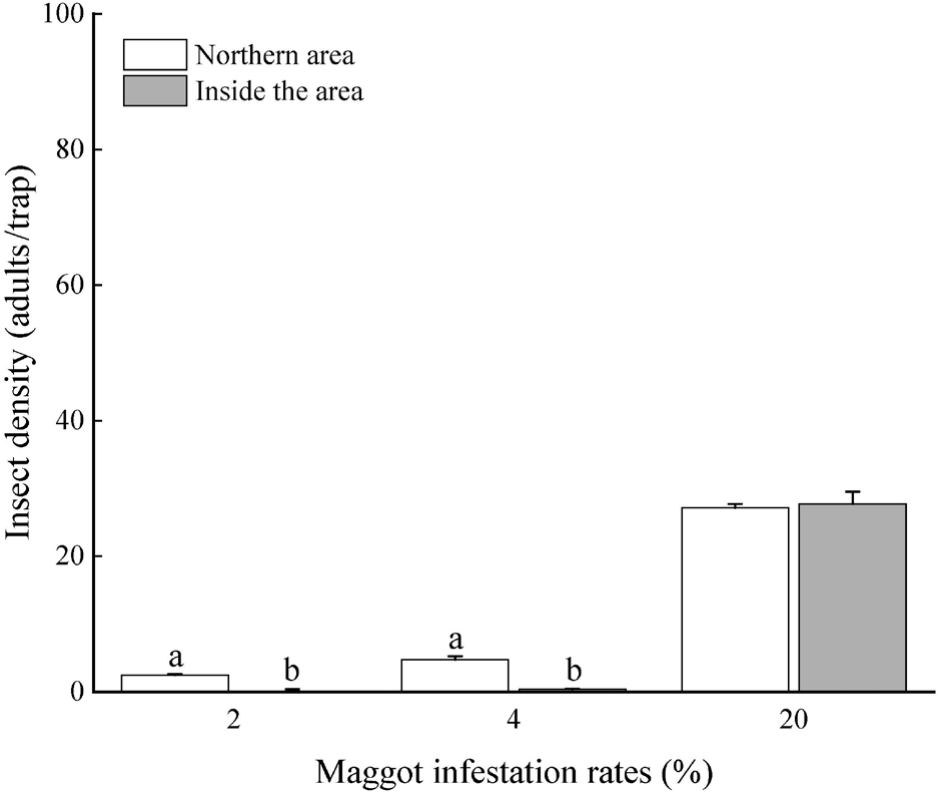
The density of *B. minax* in the north and interior of the orchard under different infestation rates. Different lowercase letters indicate significant differences in density between the northern and inner parts of the orchard. (*P*<0.05).

When the maggot infestation rates were 2% and 4%, the average adult trap catches in the northern region were 2.5 and 4.8 adults per trap, respectively, which were significantly higher than those in the inner part (2% infestation rate: 0.1 adults per trap, *U* = 0.000, *W* = 15.000, *Z* = −2.730, *P* = 0.008; 4% infestation rate: 0.4 adults per trap, *U* = 0.000, *W* = 15.000, *Z* = −2.785, *P* = 0.008). However, at a maggot infestation rate of 20%, there was no significant difference in the density of *B. minax* adults between the northern and inner parts of the orchard (*t* = −0.614; *P* = 0.567), the average adult trap catches had densities of 27.0 and 27.7 adults per trap, respectively (Fig. 4).

### The Percentages of Insects at Different Distances

**Fig. 5.**
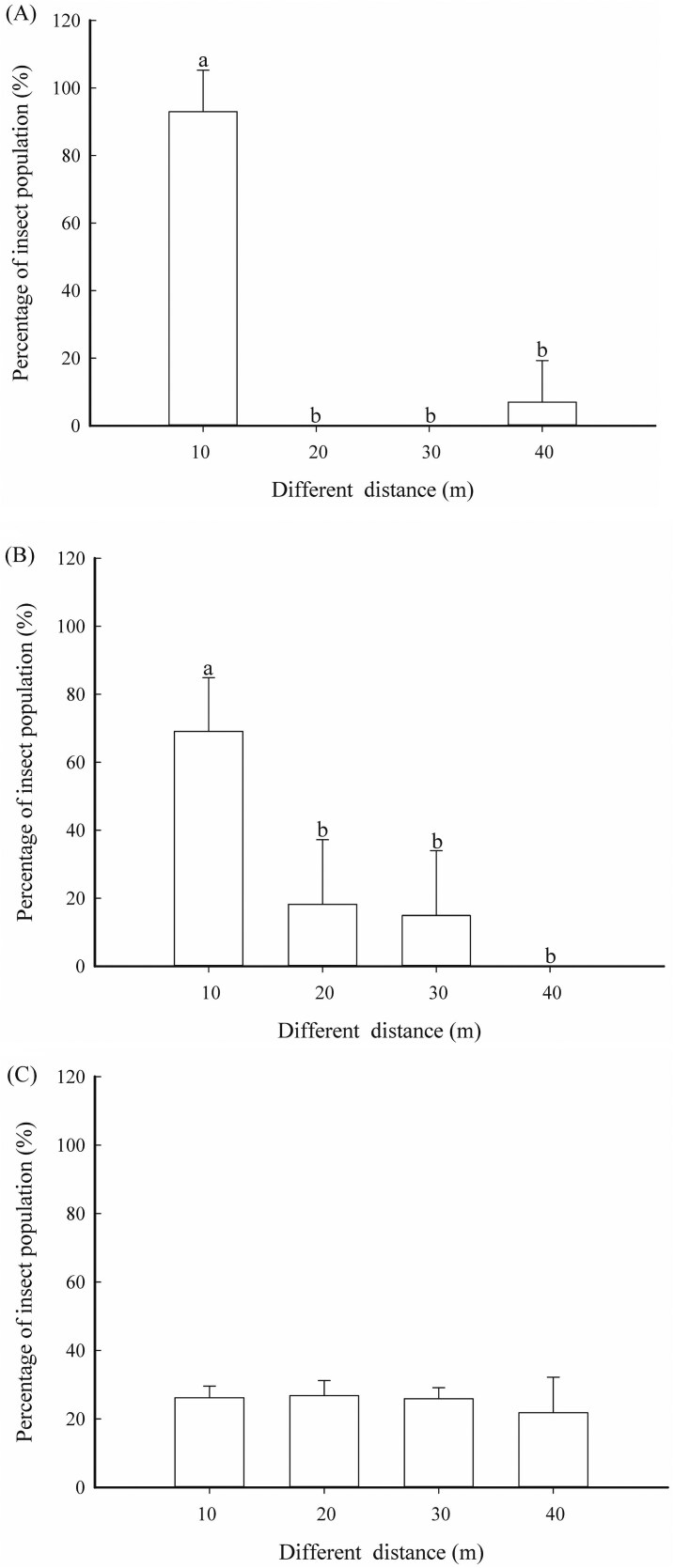
The percentage of the *B. minax* population at varying distances from the side with noncitrus trees under different densities. A) 2% infestation rates, B) 4% infestation rates, and C) 20% infestation rates. Different lowercase letters indicate significant differences in insect population percentages at different distances within the same density (*P*<0.05).

When the maggot infestation rate was 2%, the percentages of *B. minax* adults at 10 m from noncitrus trees (93.0%) was significantly higher than at 20 m (0%), 30 m (0%), and 40 m (7.0%) (*F* = 111.698; df = 3, 16; *P* < 0.001). At a 4% infestation rate, the percentages of adults at 10 m (69.1%) were also significantly higher than at 20 m (18.2%), 30 m (14.9%), and 40 m (0%) (*F* = 15.087; df = 3, 16; *P* < 0.001). However, at a 20% infestation rate, there was no significant difference in the percentages of *B. minax* adults at 10, 20, 30, and 40 m from noncitrus trees (*F* = 0.937; df = 3, 16; *P* = 0.45), with percentages of 26.2%, 26.8%, 25.9%, and 21.8%, respectively (Fig. 5).

## Discussion

We measured fruit fly density using maggot infestation rates. The results indicate that when *B. minax* density is low, adults exhibit a strong edge effect, with concentrations primarily along the side of the citrus orchard adjacent to noncitrus trees. When the *B. minax* density is low, adults exhibit a strong edge effect, with concentrations primarily along the side of the citrus orchard adjacent to noncitrus trees. However, at higher densities, *B. minax* adults are evenly distributed throughout the entire orchard. After *B. minax* emerging, adults immediately migrate to feed on noncitrus trees outside the orchard. For about 14 to 17 d post-emergence, they experience a resting period on these trees, during which no *B. minax* adults are captured within the orchard (Li et al. 2018, Wang 2018, He et al. 2019, Xu et al. 2023). When the density of *B. minax* adults is low, the mixed forest area at the orchard’s edge provides sufficient resources for newly emerged adults to rest and feed. This allows them to reach sexual maturity, gaining the ability to mate and lay eggs. Consequently, the edge zone between the orchard and the forest becomes the area with the highest probability of adult emergence, characterized by a pronounced marginal effect (Li et al. 2012, Wang et al. 2016). As the density increases, there is a greater demand for mating and egg-laying resources. Due to the strong flying ability of *B. minax*, adults are capable of dispersing throughout the orchard, and density can influence the spatial distribution pattern (Wang et al. 1990, 2002). Consequently, when the insect density is high, *B. minax* adults are evenly distributed across the entire orchard.

Many insects exhibit edge distribution effects, and understanding these effects—such as those observed in the distribution of *B. minax* adults—can help optimize pest detection, pesticide application, and the release of natural enemies (Nguyen and Nansen 2018). *Aphis fabae* Scop primarily inhabits the edges of fields, gradually spreading throughout the area; however, the field’s perimeter remains the zone with the highest aphid density (Winder et al. 1999). Similarly, compared to the interior of the orchard, the activity of *Osmia bicornis* L. is more pronounced at the orchard’s edge (Gruber et al. 2011). When *Lygus hesperus* adults first migrate into lentil fields, they are distributed in clusters. By the middle of the growing season, at low insect population densities, *L. hesperus* adults remain aggregated, while at high insect densities, the distribution of *L. hesperus* adults is more uniform or random (Schotzko and O’Keefe 1989).

In pest control, understanding insect population density is crucial to avoid significant waste of labor, pesticides, and control costs. Additionally, knowledge of insect distribution can further reveal their ecological characteristics, thereby enhancing the effectiveness of control measures. Our observations explain the different distribution patterns of *B. minax* adults at low and high insect densities. Understanding the aggregation distribution of *B. minax* adults at different density is crucial for the timely and effective control of *B. minax* adult infestation. When the densities of *B. minax* adults in the field is low, the proportion of insects captured at attraction points 10 to 20 m from the noncitrus trees side can reach 66.9% to 93.0%. In this case, controlling the flies by concentrating attraction points only on orange trees 10 to 20 m from the noncitrus trees side can prevent the waste associated with setting traps throughout the entire orchard and also save labor. However, when the density is high, *B. minax* adults are evenly distributed throughout the entire orchard, necessitating the placement of traps across the whole area.
